# Tubularized incised plate urethroplasty for distal hypospadias: A literature review

**DOI:** 10.4103/0970-1591.40619

**Published:** 2008

**Authors:** Luis Henrique P. Braga, Armando J. Lorenzo, Joao L. Pippi Salle

**Affiliations:** Division of Urology and University of Toronto, The Hospital for Sick Children, Toronto, ON, Canada

**Keywords:** Hypospadias, Snodgrass procedure, tubularized incised plate repair, tubularized incised urethroplasty

## Abstract

The tubularized incised plate (TIP) urethroplasty or Snodgrass procedure has gained worldwide acceptance for distal hypospadias repair due to its low complication rate, good cosmetic result, and technical simplicity. As a result, several articles have been published concerning various aspects and subtle variations of this procedure. The aim of this review is to critically and systematically analyze the published complication rates of TIP repair for distal hypospadias in children. We also reviewed the surgical modifications that have been introduced to the original technique and discussed the potential impact on the final outcome of the Snodgrass procedure.

## INTRODUCTION

The tubularized incised plate (TIP) repair is based on an old principle of urethral plate tubularization, also known as the Thiersch-Duplay procedure.[1,2] Although a good concept, its main drawback was the limitation imposed by the width of the urethral plate. Historically, if the urethral groove was not wide enough for tubularization *in situ*, alternative approaches such as the Mathieu urethroplasty (flip-flap technique) or a vascularized island flap were performed.[3–9 In 1994, Snodgrass popularized the concept of urethral plate incision with subsequent tubularization and secondary dorsal healing for primary hypospadias repair.[10] Not surprisingly, the principle of incising the urethral plate had been employed before, but for different purposes.[11,12] In 1987 Ordeszewski incised the plate to achieve easier tubularization in redo cases where the urethral plate is often scarred.[11] Two years later, Rich took advantage of hinging the plate in onlay island flap repairs in order to improve the configuration of the meatus.[12]

This relatively simple yet elegant and effective procedure has gained widespread acceptance since its description 13 years ago, currently being recognized as the surgical technique of choice for distal hypospadias, according to a recent survey of Pediatric Urologists.[13]

In this article, we critically and systematically compared the complication rates of TIP repair for distal hypospadias in children. We also reviewed the surgical modifications that have been introduced to the original technique, commenting on how they have potentially affected the final outcome of the Snodgrass procedure.

## METHOD OF LITERATURE REVIEW

We established inclusion criteria for the articles prior to the literature search. A review of the English literature was performed via the database MEDLINE/Pubmed from January 1994 to September 2007 using Medline Subjects Headings (MeSH) hypospadias and TIP urethroplasty or hypospadias and ℌSnodgrass repairℍ. Full text hard copies of relevant abstracts were retrieved. Full text articles then underwent secondary review and only articles that addressed the use of TIP repair for distal hypospadias in children were included. Studies related to the use of TIP urethroplasty for mid-shaft and proximal hypospadias were excluded as well as review articles and those for reoperations and animal studies. The bibliographies of all relevant articles were reviewed for other missed pertinent citations. Overall complication rate included fistula, meatal stenosis, dehiscence, recurrent ventral curvature, and hematoma requiring reintervention. Descriptive statistics were performed using SPSS software version 15. Complication rates between various series were compared using two-sided χ^2^-test.

## RESULTS

A total of 141 studies were identified in our MEDLINE/Pubmed review. Of these articles, 44 were discarded because they were not related to hypospadias surgery. Then, 97 remaining studies provided data on TIP repair for hypospadias. Of these, 31 did not meet the inclusion criteria as those studies focused exclusively on mid-shaft and proximal hypospadias (*n* = 8), [14–21] reoperation or staged repair (*n* = 11), [11,22–31] experimental work (*n* = 4) [32–35] or reviewing the technique (*n* = 8). [36–43]

**Figure 1:**
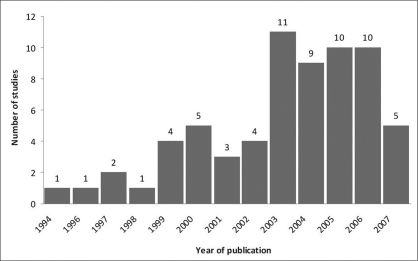
Distribution of studies involving TIP repair for distal hypospadias according to year of publication

A total of 66 articles involving TIP repair for distal hypospadias were included in our analysis and are displayed by year of publication in Figure 1. Seven studies involved comparison between the Snodgrass and Mathieu techniques and are presented Table 1.[3–6,44–46] Other seven articles introduced modifications to the original TIP urethroplasty and are presented Table 2. [47–53] Fifteen studies addressed distal and proximal defects [51,54–67 [Table 3] and seven reported on primary and secondary TIP repairs[3,53,62,64,66,68,69] [Table 4]. As a result, only primary distal cases were selected from those studies and added to the overall analysis. In aggregate, these 66 studies included 4554 children undergoing the Snodgrass technique exclusively for distal hypospadias. Excluding four articles that described the use of TIP urethroplasty in adults,[65,70–72] the median age was 22.9 months or 1.9 years (mean = 32.1 months or 2.7 years), ranging from 6 months to 18 years and the median follow-up was 16.5, ranging from 1 to 120 months.

**Table 1:** Studies comparing the Snodgrass repair to the Mathieu technique in children with distal hypospadias

Author	Year	*n* (Snodgrass/Mathieu)	Fistula rate (%)	
			
			Snodgrass	Mathieu
Oswald[6]	2000	60 (30/30)	3.3	6.6
Imamoglu[3]	2003	55 (32/33)	12.5	12.1
Guo[5]	2004	79 (36/43)	8.3	25.6
Vervederis[45]*	2005	20 (10/10)	-	-
Moradi[44]	2005	33 (15/18)	13	5.6
Anwar[4]**	2006	90 (45/45)	-	-
Germiyanolu[46]	2006	117 (76/41)	16	7

* Study reported on cosmetic evaluation by a panel

** Mathieu procedure was associated with more complications

**Table 2:** Studies reporting on technical modifications for the Snodgrass procedure in patients with distal hypospadias

Author	Year	*n*	Modification	Fistula rate (%)
Kolon[53]*	2000	11	Dorsal inlay graft	6.3
Furness[51]	2003	106	Ventral dartos flap	1.0
Soygur[50]	2004	60	Ventral dartos flap	8.3
Al-Hunayan[48]	2005	83	Lateral skin flap	4.8
Jayanthi[49]	2005	110	Suturing proximally	1.0
Kiss[52]	2006	19	Mathieu + plate incision	5.2
Baccala[47]	2006	85	Local de-epithelialized skin flap	2.3

* Study also involved redo hypospadias

**Table 3:** Studies reporting on distal and proximal hypospadias*

Author	Year	*n*	Mean age (range) months	Follow-up (range) months	Overall complication rate (%)	Fistula (%)	Meatal stenosis (%)
Castellan[56]*	2000	70	-	24	4.1	-	-
Guralnick[54]*	2000	28	18(7–72)	9(2–20)	27.2	16.2	5.4
Elbakry[58]*	2002	52	(24–216)	28(6–52)	10	-	-
Cheng[55]	2002	414	-	(4–66)	0.2	0	0.2
Samuel[61]	2003	65	14(6–22)	4	6	5	0
Furness[51]	2003	106	21(5–192)	14(3–38)	1	1	0
Sozubir[67]	2003	75	20(3–432)	9	4	4	0
Elicevik[59]	2004	324	52(24–168)	(6–60)	23	15	7
Chartterjee[60]	2004	25	55(12–264)	24(12–48)	7.5	7.5	0
El-Sherbiny[62]	2004	106	84(12–264)	10(5–15)	11	10	1
Djordjevic[57]	2005	51	(12–132)	21(6–65)	0	0	0
Mustafa[64]*	2005	12	(12–216)	-	33.4	25	8.4
Sharma[65]	2005	5	(216–312)	(3–36)	20	20	0
Kockvara[66]	2005	72	(13–204)	28	11	-	-
Asanuma[63]	2007	19	21(14–55)	22	3.6	3.6	0

* Studies only provided the combined complication rate for distal and proximal hypospadias

**Table 4:** Studies reporting on primary and secondary (redo) distal hypospadias*

Author	Year	*n*	Mean age (range) months	Follow-up (range) months	Overall complication (%)	Fistula (%)	Meatal stenosis (%)
Retik[69]	1998	27	8.5(5–312)	9(4–14)	3.3	3.3	0
Kolon[53]*	2000	11	16(6–120)	21(3–37)	6.3	-	-
Riccabona[68]	2003	168	21(6–204)	42(5–71)	6.9	5.3	0
Imamoglu[3]*	2003	32	70(36–204)	24	21.4	-	-
El-Sherbiny[62]	2004	106	84(12–264)	10(5–15)	11	10	1
Kocvara[66]	2005	72	-(13–204)	28	11	-	-
Mustafa[64]*	2005	12	-(12–216)	-	33.4	25	8.4

* Studies included overall complication rate for both primary and secondary cases

### Overall complication rate

The overall complication rate for the Snodgrass procedure ranged from 0 to 50%. The highest complication rate was found in a study that involved only two patients with distal hypospadias, aged 14 and 62 years. The older patient in this series developed a fistula explaining the high (50%) complication rate. [71] This study also described the experience with other techniques for hypospadias repair in adults and concluded that performing hypospadias surgery in older patients was associated with more complications. O'Connor also included three adults (oldest being 39 years of age) in his series, but did not discriminate the complications in this particular subgroup.[70] Likewise, Sharma reported on 13 adults aged 18–26 years of age who underwent TIP repair. Five of them had distal hypospadias and only one developed a fistula (20%).[65] The largest study addressing hypospadias in adults included 97 patients, but only 14 underwent TIP urethroplasty. The overall complication rate was 8.7%, but it was not possible to separate the complications involving only TIP repairs.[72] Therefore, even after combining data from these four studies, limitations attributable to the small sample size (*n* = 24) do not allow one to conclude that TIP repair has a higher complication rate in adults.

If we exclude these four articles that included adult patients, the overall complication rate of TIP urethroplasty goes down to 33%. Here again, some points are worth mentioning. If we exclude another eight studies (for reasons that will be explained below), the complication rate drops even further, reaching 23%.[3,5,44,54,64,73–75] The high complication rates from the Thailand[75] (33%) and the Nairobi[74] (30%) studies may be explained by the lack of familiarity with the surgical technique as both articles described their initial experiences with the Snodgrass procedure. For instance, the Nairobi study was able to reduce their 76% complication rate using other techniques for hypospadias repair to 30% after adopting TIP urethroplasty.[74]

Furthermore, four articles that reported high overall complication rates with TIP repair for distal hypospadias included primary and redo cases.[3,54,64,73] Similar results have been reported by other studies in the literature showing that redo hypospadias are associated with more complications.[22–31] Unfortunately, we could not determine the isolated complication rate for the primary cases based on the data provided. Therefore, what has been presented for those four studies was the combined complication rate for both primary and secondary hypospadias, explaining the high figures.

Finally, two other studies with higher than expected complication rates deserve to be cited. They both involve comparison between the Mathieu and the Snodgrass techniques. These studies probably reflect a change in practice, switching from a traditional operation (Mathieu) to a new technique (Snodgrass), explaining the high complication rates observed during their initial experience with TIP urethroplasty. One article showed that TIP repair was associated with a lower complication rate;[5] however, the other demonstrated that fistulas occurred less often after the Mathieu procedure rather than following the Snodgrass technique.[44]

So far, we have excluded 12 articles from our initial 66 studies. As a result, the median overall complication rate was 6.5% (mean = 7.3%), ranging from 0 to 23% in the remaining 54 studies. Of these, 15 involved distal and proximal defects[51,54–67] and seven included primary and redo cases.[3,53,62,64,66,68,69] Due to the clinical heterogeneity and in order to avoid misinterpretation of the data, only primary distal hypospadias were selected from those studies. The mean and median overall complication, fistula, and metal stenosis rates were calculated based on the data available in the remaining 54 articles.[3–6,10,44–70,73–94]

**Figure 2:**
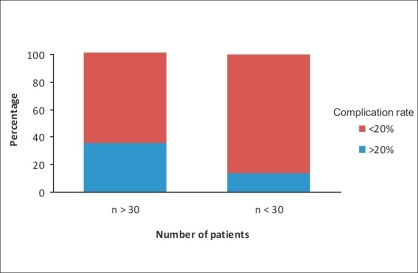
Comparison of complication rates between studies including large (*n* > 30) and small (*n* < 30) number of patients

It is noteworthy to mention that studies with a small sample size (*n* < 30) reported more complications (>20%) in comparison to studies involving large number of patients (74.5% vs. 46.2%, *P* = 0.05) as shown in Figure 2. This suggests that surgeon experience and high volume of cases may contribute to reduce the number of complications in any series, although some of these studies included multisurgeon practice.

In this setting, Snodgrass' own practice supports the importance of surgeon experience and case-load. When one analyzes only his results with distal hypospadias, his overall complication rate ranges from 0 to 7%.[10,67,85,87,90,95–99] as seen in Table 5. The 7% figure though was the result of a multicenter study involving five different centers across the United States and one in Europe,[96] not a single surgeon practice. If we only consider his single experience, the reported complication rate varies from 0 to 4%, mostly fistulas and very few meatal stenoses (approximately 2%).

**Table 5:** Snodgrass' articles on distal hypospadias

Year	*n*	Mean age (range) months	Man follow-up (range) months	Overall complication (%)	Fistula (%)	Meatal stenosis (%)
1994[10]	16	(6–132)	22	0	0	0
1996[96]	148	-	-	7	5	2
1999[99]¶	72	-	12	-	-	-
1999[95]*	62	20(5–192)	15(6–48)	-	-	0
2000[87]§	13	Prepubertal	-	-	-	-
2002[90]**	108	-	10.5(1.5–100)	-	-	3
2003[67]∞	75	20(3–432)	9	4	4	0
2004[85]	159	20(3–144)	8	2	2	0
2006[97]δ	46	14(6–84)	24(5–60)	4	-	-
2006[98]π	51	30.8(4–143)	8(2–48)	2	2	0

Studies reported on: ^§^histology of the urethral plate

¶ suture tracks

* urethral calibration

** comparison of meatal dilatation between 2 groups

δ foreskin reconstruction

π after previous circumcision

∞ distal, midshaft and penoscrotal hypospadias

### Fistula rate

Of 54 studies reporting on occurrence of fistulas, the median fistula rate was 5.0% (mean = 5.9%), ranging from 0 to 16%. [3–6,10,44–70,73–94] The Hospital for Sick Children experience included 48 children who underwent TIP urethroplasty from 1996 to 2000, representing the early part of our experience with this technique. [94] The fistula rate was 4%, similar to the figures summarized with this review. Several factors may influence fistula formation: surgical technique, delicate tissue handling, patient age, type of hypospadias defect, surgeon experience, waterproof urethroplasty coverage, and concomitant foreskin reconstruction, among others.[43] In this review, we have found five articles involving TIP repair associated with foreskin preservation.[73,80,97,100] In the largest series published on this topic, involving 149 children with distal hypospadias, Leclair *et al.* reported similar fistula rates for patients with and without prepuce preservation.[80] In contrast, our preliminary analysis suggests a higher fistula rate (14%) in children with foreskin preservation when compared to those cases where the foreskin was removed and a dartos flap was harvested and transferred ventrally. We speculate that this might have occurred due to lack of waterproof coverage (dartos flap) in children who had foreskin preservation. [101]

Surgical principles are important, especially in hypospadias surgery. With that in mind, Snodgrass has recommended two-layer neourethra closure to decrease fistula formation in all types of hypospadias defects.[16] He has reported that his fistula rate reduced from 33 to 11% when performing two-layer urethroplasty in proximal hypospadias. Careful interpretation of his results has shown an unbalanced distribution of other technical factors (confounders) between the assembled groups which were not accounted for and might have affected the outcome.[16] Similarly Cheng *et al.* reported <1% complication rate for distal hypospadias in more than 400 patients in whom the urethroplasty was performed in two layers.[55] Despite these excellent results, no prospective comparative study involving one vs. two layers has been conducted to date. According to Snodgrass's experience, the fistula rate reduced to almost 0% when in addition to two-layer neourethra closure, the urethroplasty was covered with a tunica vaginalis flap instead of a dartos flap.[102]

Age at operation has also been suggested to affect the outcome of hypospadias surgery.[103] Perlmutter *et al.* have reported on 194 boys who underwent TIP repair for distal hypospadias and concluded that the fistula rate was significantly lower in children younger than 6 months vs. older patients (>6 months).[103] These findings support the current tendency of early hypospadias repair in children, normally between 6 and 18 months of age.[43]

### Meatal Stenosis

The mean meatal stenosis rate was 2.1% (median = 0%), varying from 0 to 17% in 53 studies. [3–6,10,44,45,47–70,73–92,94] In contrast, other series have shown a surprisingly high rate of meatal stenosis, ranging from 6 to 20%.[44,48,50,64,73–75,77,80] This has been a controversial topic and considered to be possibly related to the surgical technique (i.e., carrying the urethral plate incisions far too distal), as the drawings from the original technique implied that the urethral tubularization should include all the extension of the incisions to the tip of the glans. The high rates of meatal stenosis may reflect strict adherence to this description by Snodgrass. On the other hand, Snodgrass has reported meatal stenosis rates below 1% and has demonstrated with calibration and urethroscopy that the neourethra lumen after TIP repair is adequate and allows introduction of a 10-Fr feeding tube.[95] He has also shown that re-epitheliazation occurs by second intention after incision of the urethral plate.[40] These findings support the thinking that urethral strictures or meatal stenosis should not occur after TIP repair for distal hypospadias, as long as the surgeon does not tubularize the incisions in the urethral plate too distally into the glans.

### Technical Modifications

Most technical modifications of the Snodgrass technique have included different ways to harvest the dartos flap in order to cover the urethroplasty, not changes to the urethroplasty technique itself as summarized in Table 2. In this setting, the variations described include a local de-epithelialized skin flap,[47] a lateral skin flap, [48] or a ventral-based dartos flap.[50,51] One urethroplasty modification was proposed by Jayanthi, when he suggested that the tubularization should be performed over a 10- or 12-Fr feeding tube and working from the meatus proximally.[49] Another surgical change was described by Kiss who decided to incise the urethral plate while performing a Mathieu type of repair[52] but, perhaps the most creative innovation has been the ℌ*Snodgraft*ℍ procedure which consists in covering the raw surface of the incised plate with an inlay preputial or buccal graft. Some authors have utilized this technique preferably for redo hypospadias,[30,53,104 but its application in primary cases has also been described.[63] In 1998, Kolon and Gonzales were the first to describe the use of dorsal inlay graft urethroplasty for redo cases.[53] Following them, Haynes and Malone in 1999 and then Schwentner and colleagues in 2006 applied the same technique for salvage hypospadias repair.[30,104] Recently, Asanuma *et al.* published on 28 children who underwent dorsal inlay graft urethroplasty for primary hypospadias (17 distal and 9 midshaft/proximal) and achieved good results with an acceptable fistula rate (3.6%).[63]

### Comparative Analysis Between Mathieu and Snodgrass Techniques

We were able to find seven studies in our literature review that compared the Snodgrass repair to the Mathieu technique[3–6,44–46 as shown in Table 1. Four of them reported fewer complications with the TIP repair.[3–5,44] Two studies showed no difference between the two procedures in regards to complications, but stated that the Snodgrass technique seemed to achieve better cosmetic results.[45,46] In the only prospective trial encountered in this review, Oswald *et al.* randomly allocated 30 children to undergo the Snodgrass operation and 30 to have the Mathieu procedure. The authors concluded that TIP repair was associated with lower complication rates vs. the Mathieu technique and that the cosmetic results were far more satisfactory with the TIP operation.[6]

## CONCLUSION

We were able to identify only one prospective randomized study involving TIP repair for distal hypospadias in children.[6] Future efforts should be made to start prospective data collection and initiate randomized clinical trials involving hypospadias surgery. As with any review, clinical heterogeneity of the studies due to different geographic locations as well as singular demographic factors offered some limitations to the comparability of the data. Furthermore, confounders were not always accessible due to ambiguity in reporting among authors, who combined distal as well as proximal hypospadias and primary and redo cases. Both study variability and confounding factors may have affected the validity and therefore the generalizability of this review.

Thus far, TIP urethroplasty appears to be the best available procedure for correction of distal hypospadias in children. By using standard techniques cited in this review, the pediatric urologist can expect a predictable outcome with complications rates below 10%.
